# Characterization of Water Extractable Arabinoxylans from a Spring Wheat Flour: Rheological Properties and Microstructure

**DOI:** 10.3390/molecules18078417

**Published:** 2013-07-16

**Authors:** Adriana Morales-Ortega, Elizabeth Carvajal-Millan, Yolanda López-Franco, Agustín Rascón-Chu, Jaime Lizardi-Mendoza, Patricia Torres-Chavez, Alma Campa-Mada

**Affiliations:** 1 Laboratory of Biopolymers, CTAOA. Research Center for Food and Development, CIAD, A.C. Carretera a La Victoria Km. 0.6, Hermosillo, Sonora 83304, Mexico; E-Mails: adriana.morales@estudiantes.ciad.mx (A.M.-O.); lopezf@ciad.mx (Y.L.-F.); jalim@ciad.mx (J.L.-M.); acampa@ciad.mx (A.C.-M.); 2 Laboratory of Biotechnology, CTAOV. Research Center for Food and Development, CIAD, A.C. Carretera a La Victoria Km. 0.6, Hermosillo, Sonora 83304, Mexico; E-Mail: arascon@ciad.mx; 3 Department of Food Research and Graduate Program (DIPA), University of Sonora, Hermosillo, Sonora 83000, Mexico; E-Mail: pitorres@guayacan.uson.mx

**Keywords:** ferulated arabinoxylans, gels, ferulic acid, diferulic acid

## Abstract

In the present study water extractable arabinoxylans (WEAX) from a Mexican spring wheat flour (cv. Tacupeto F2001) were isolated, characterized and gelled and the gel rheological properties and microstructure were investigated. These WEAX presented an arabinose to xylose ratio of 0.66, a ferulic acid and diferulic acid content of 0.526 and 0.036 µg/mg WEAX, respectively and a Fourier Transform Infra-Red (FT-IR) spectrum typical of arabinoxylans. The intrinsic viscosity and viscosimetric molecular weight values for WEAX were 3.5 dL/g and 504 kDa, respectively. WEAX solution at 2% (w/v) formed gels induced by a laccase as cross-linking agent. Cured WEAX gels registered storage (G’) and loss (G’’) modulus values of 31 and 5 Pa, respectively and a diferulic acid content of 0.12 µg/mg WEAX, only traces of triferulic acid were detected. Scanning electron microscopy analysis of the lyophilized WEAX gels showed that this material resembles that of an imperfect honeycomb.

## 1. Introduction

The improvement of yield and quality of wheat (*Triticum aestivum* L.) varieties are focused on meeting current and future demands for grains. To achieve this objective, breeding programs need a thorough understanding of the constituents of grain as the biochemical constituents of wheat grain largely determine its end-use quality [1]. Tacupeto F2001 is a spring wheat variety developed by The International Maize and Wheat Improvement Center (CIMMYT) in Mexico, and provided to National Institute for Investigation in Forestry, Agriculture and Animal Production (INIFAP) for testing and release. INIFAP released this wheat variety for cultivation in Northwestern Mexico. Tacupeto F2001 is a bread wheat variety presenting resistance to leaf rust. Previous studies have examined the quality parameters of Tacupeto F2001 wheat [2,3,4]. However, to our knowledge, there are no reports about Tacupeto F2001 arabinoxylans, which are key constituents of wheat grain playing an important role on the grain functionality. Arabinoxylans are important cereal non-starch polysaccharide constituted of a linear backbone of α-(1→4)-linked D-xylopyranosyl units to which α-L-arabinofuranosyl substituents are attached through O-2 and/or O-3. Some of the arabinose residues are ester linked on (O)-5 by ferulic acid (FA) (3-methoxy-4-hydroxycinnamic acid) [5]. These polysaccharides have been classified as water extractable (WEAX) or water-unextractable (WUAX). WEAX form highly viscous solutions and gel through ferulic acid covalent cross-linking upon oxidation by some chemical or enzymatic free-radicals generating agents [6]. Laccase (*p*-diphenol oxygen oxidoreductase, EC 1.10.3.2), blue multi-copper enzymes of white rot fungi oxidizes FA from WEAX resulting in the formation of five different di-FA structures (5-5′-, 8-5′-benzo-, 8-O-4′-, 8-5′- and 8-8′-di-FA), the 8-5′ form being always preponderant, and a tri-FA [7,8]. These covalent WEAX cross-links have been commonly considered as responsible of the WEAX network development even if weak interactions also contribute to the final gel properties [9]. Most of the polysaccharide gels are stabilized by physical interactions (hydrogen bonding and/or ionic interaction); polysaccharide covalent networks such as WEAX gels are not very common. Covalently cross-linked gels are generally strong, form quickly, are not temperature dependent and exhibit no syneresis after long time storage. Furthermore, WEAX and WEAX gels have interesting functional properties, which have not been exploited even though the WEAX neutral taste and odor are desirable properties for industrial applications. WEAX networks have a high water absorption capacity (up to 100 g of water per gram of polymer) and they are not sensible to electrolytes or pH [5,6].

Understanding the amount of WEAX that is extractable from Tacupeto F2001, and the characteristics of these WEAX and the WEAX gels formed can be useful to wheat-breeding programs where the aim is to develop wheat cultivars with superior and consistent endues quality. The objective of this study was to investigate for the first time the physico-chemical characteristics of WEAX extracted from Tacupeto F2001 wheat grain as well as their gelling capability and the gel rheological properties and microstructure. 

## 2. Results and Discussion

### 2.1. Extraction and Characterization of WEAX

WEAX have been extracted from 10 kg of Tacupeto F2001 wheat flour. Yield of WEAX extracted from wheat flour was 0.50% (w/w) on a dry matter basis (db, w WEAX/w wheat flour). Similar WEAX yield values have been reported for flours of different wheat varieties [9,10,11]. WEAX composition is presented in Table 1. The arabinoxylan content of the extract was estimated from the sum of xylose + arabinose. The arabinoxylan content was 63% db, which is close to the value reported for other wheat WEAX [9]. A residual amount of glucose was also quantified. The FA content (0.526 μg/mg WEAX) was in the range reported for other wheat WEAX [7,8,10,12]. Small levels of di-FA have been detected in WEAX (0.0326 μg/mg WEAX) suggesting that some arabinoxylan chains might be cross-linked as previously reported [13,14]. The percentages of each one of the different di-FA presents in the WEAX were: 81, 17, and 2% for the 8-5′ (mainly in the benzofuran form), 8-O-4′, and 5-5′ structures, respectively. The 8-8′ dehydrodimer was not detected in this study. The predominance of 8-5′ and 8-O-4′ di-FA structures has also been reported in arabinoxylans from wheat and barley flour [14]. The tri-FA 4-O-8′, 5′-5′′ was detected only in traces. The degree of substitution (arabinose to xylose ratio, 0.66) was characteristic of wheat endosperm arabinoxylans (0.53–0.7) [8,10,15]. The intrinsic viscosity ([η]) and viscosimetric molecular weight (Mv) values were 3.5 dL/g and 504 kDa, respectively, which are in the range previously reported for other WEAX wheat [5,6].

**Table 1:** Composition of water extractable arabinoxylans.

Arabinose ^a^	23.50 ± 1.1
Xylose ^a^	35.30 ± 0.4
Glucose ^a^	4.80 ± 0.4
Protein ^a^	4.20 ± 0.01
Ferulic acid ^b^	0.526 ± 0.001
Diferulic acids ^b^	0.036 ± 0.001
Triferulic acid ^b^	traces

^a^ Results are expressed in g/100 g WEAX dry matter; ^b^ Phenolics are expressed in µg/mg WEAX dry matter; All results are obtained from duplicates.

**Figure 1:**
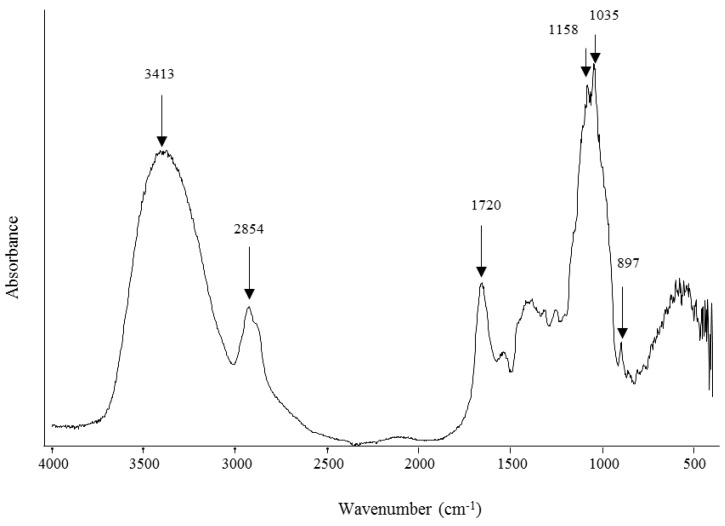
FT-IR spectrum of water extractable arabinoxylans.

The Fourier transform infrared (FTIR) spectrum of WEAX is presented in Figure 1. This figure shows mainly a broad absorbance band for polysaccharides at 1200–800 cm^–1^. The main band centered at 1,035 cm^–1^ could be assigned to C-OH bending, with shoulders at 1158, and 897 cm^–1^ that were related to the antisymmetric C-O-C stretching mode of the glycosidic link and β(1-4) linkages [16]. The region from 3500 to 1800 cm^−1^ is the fingerprint region of polysaccharides related to arabinoxylans, with two bands (3,413 cm^−1^ corresponding to stretching of the OH groups and 2854 cm^−1^ corresponding to the CH_2_ groups) [17]. An absorbance band was observed at 1720 cm^−1^ implying a low degree of esterification with aromatic esters such as ferulic acids [18].

**Figure 2:**
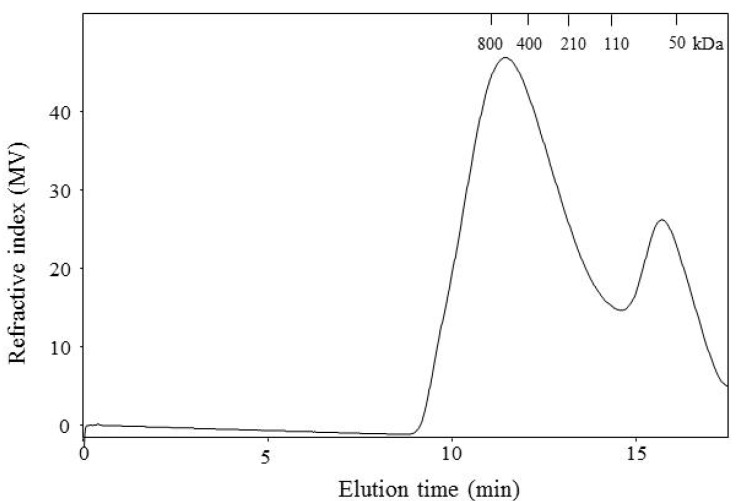
Size-exclusion HPLC elution profiles of WEAX. Retention time of pullulan standards with molecular weight varying from 50 to 800 kDa are indicated.

Figure 2 shows the elution profile and molecular weight distribution of WEAX. The extracted polysaccharide was clearly polydisperse with apparent molecular weights in a broad range. The elution pattern of WEAX shows that this material comprised two populations of polysaccharides that differed considerably in hydrodynamic volumes. A major peak was registered at high molecular weight region (retention time between 9 and 14 min). A shoulder to the right of the major peak, at low molecular weight region, was registered indicating a second, low molecular weight polysaccharide chain population in this preparation. A similar behavior has been previously reported for arabinoxylans isolated from the flours of wheat cultivars [19,20]. The two peaks registered in the present study were near to the retention time of pullulan standards with molecular weight varying from 50 to 800 kDa (Figure 2). 

### 2.2. WEAX Gelation

**Figure 3:**
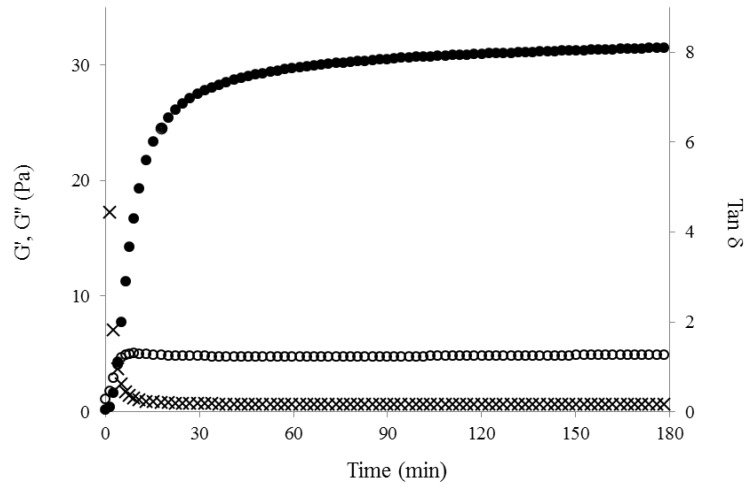
Rheological kinetics of 2% (w/v) WEAX solution gelation by laccase. G′ (○), G′′ (●), tan δ (×). Measurements at 25 °C, 1 Hz and 10% strain.

**Figure 4:**
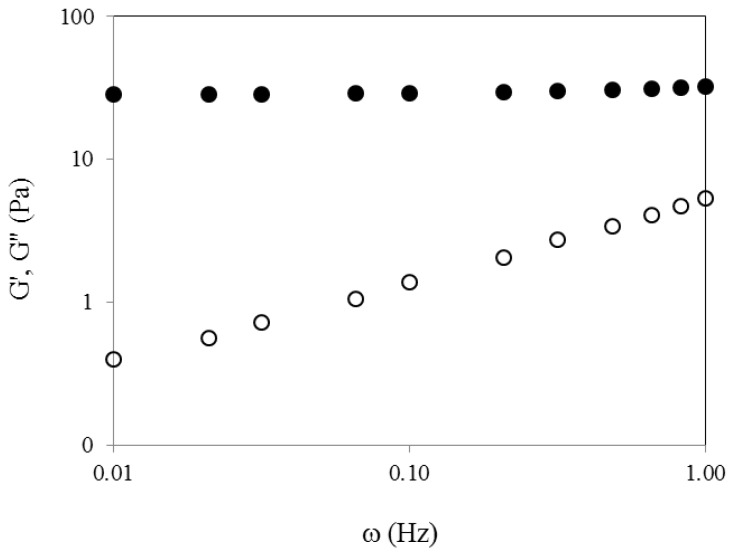
Mechanical spectrum of WEAX gel at 2% (w/v). G′ (○), G′′ (●). Measurements at 25 °C, 1 Hz and 10% strain.

The cross-linking process of WEAX was rheologically investigated by small amplitude oscillatory shear. Figure 3 shows the development of G′ and G′′ moduli versus time of 2% (w/v) WEAX solution undergoing oxidative gelation by laccase. Storage (G′) and loss (G′′) moduli rise to reach a pseudo plateau region. The final G′ and G′′ values of 2% (w/v) were 31 and 5 Pa, respectively. The gelation time (tg), calculated from the crossover of the G' and G" curves (G' > G") was 4 min. The tg value indicates the sol/gel transition point and at this point G' = G" or tan δ = G''/G' = 1 [21]. The mechanical spectra of WEAX after 2 h gelation (Figure 4), was typical of solid-like materials with a linear G′ independent of frequency and G′′ much smaller than G′ and dependent of frequency [22]. This behavior is similar to that previously reported for arabinoxylan gels cross-linked by laccase or peroxidase/H_2_O_2_ system [23,24]. During WEAX gelation ferulic acid was oxidized leading to the formation of 0.122 µg of di-FA per milligram of WEAX and only traces of tri-FA. The amounts of di-FA and tri-FA produced did not counterbalance the lost in FA. Therefore, at the end of gelation, 63% of the initial FA in the WEAX solution disappeared, with only 37% recovered as di-FA. Low ferulate recovery after oxidative treatment of arabinoxylans [7,8,23] and feruloylated sugar beet pectin [24] has been previously reported and related to the possible formation of higher oligomers of ferulate other than dimers.

### 2.3. Freeze Dried WEAX Gel

**Figure 5:**
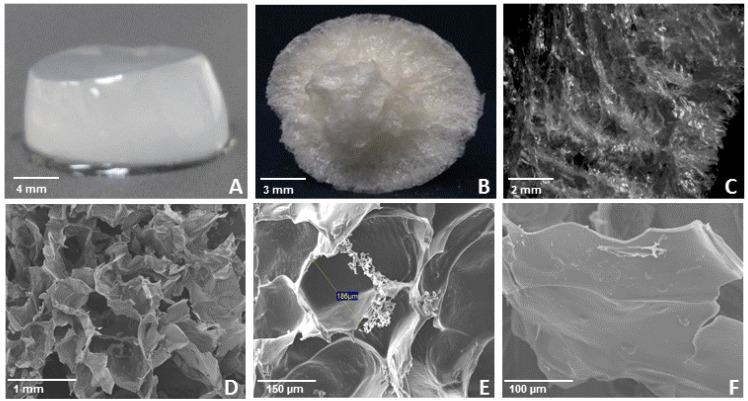
WEAX gel (**A**), lyophilized WEAX gel (**B**), stereomicrograph of lyophilized WEAX gel (**C**) and SEM micrographs of lyophilized WEAX gel at 35× magnification (**D**), 200× magnification (**E**) and 350× magnification (**F**).

In Figure 5 is shown a WEAX gel image before (A) and after freeze drying (B). Figure 5C shows a stereomicrograph of the freeze dried WEAX gel external structure. It is possible that frozen caused the crust formation of the sample. The internal structure of the lyophilized WEAX gel was observed by SEM (Figure 5D,E). The WEAX gel network presents many connections and can be compared with an irregular honeycomb structure. In the present study the average inner dimensions of the cell are approximately 200 × 400 µm (Figure 5E). This morphological microstructure is similar to that reported before for lyophilized wheat and maize AX gels [25,26,27,28]. Nevertheless, the SEM microstructure of freeze dried WEAX gels shown in Figure 5D,E is different from that recently reported for supercritical CO_2_- dried WEAX aerogels which present a more spongy network formation [29]. 

## 3. Experimental

### 3.1. Materials

Water extractable arabinoxylans (WEAX) were extracted from the endosperm of a spring wheat variety (Tacupeto F2001). Tacupeto F2001 bread wheat variety was kindly provided by a wheat milling industry in Northern Mexico (Molino La Fama). Commercial laccase (benzenediol:oxygen oxidoreductase, E.C.1.10.3.2) was from *Trametes versicolor*. All chemical reagents were purchased from Sigma Chemical Co. (St Louis, MO, USA). The grain sample was milled to pass a 0.5 mm screen using Quadrumat Sr (Branbender, South Hackensack, NJ, USA) according to the AACC method 26-10 [30]. 

### 3.2. Isolation of WEAX

WEAX were extracted from milling fraction Tacupeto F2001 (1 kg/3 L) for 15 min at 25 °C as described previously [9]. The water extract was then centrifugated (12,096 *g*, 20 °C, 15 min) and supernatant measured (2.4 L). Starch was then enzymatically degraded (amylase, 100 °C, 30 min, 2800 U/g of flour and amyloglucosidase, 3 h, 50 °C, pH 5, 24 U/g of flour). The destarched extract was deproteinized with pronase (pH 7.5, 20 °C, 16 h followed for 100 °C, 10 min, 0.4 U/g of flour). Supernatant was precipitated in 65% ethanol treated for 4 h at 4 °C. Precipitate was recovered and dried by solvent exchange (80% (v/v) ethanol, absolute ethanol and acetone) to give WEAX. Twenty extracts were prepared. 

### 3.3. Chemical and Physicochemical Analyses

#### 3.3.1. Laccase Activity

Laccase activity was measured at 25 °C from a laccase solution at 0.125 mg/mL dissolved in 0.05 M citrate-phosphate buffer pH 5.5 as previously reported [8].

#### 3.3.2. Neutral Sugar

Neutral sugar content in WEAX was determined by hydrolysis of the polysaccharides with 2 N trifluoroacetic acid at 120 °C for 2 h [9]. The reaction was stopped in ice and the extracts were evaporated under air at 40 °C, rinsed twice with 200 µL of water. The evaporated extract was solubilized in 500 µL of water. Inositol was used as internal standard. Samples were filtered through 0.2 µm (Whatman) and analysed by HPLC using a Supelcogel Pb column (300 × 7.8 mm; Supelco, Inc., Bellefont, PA, USA) eluted with 5 mM H_2_SO_4_ (filtered 0.2 µm, Whatman) at 0.6 mL/min and 50 °C. A Varian 9012 HPLC with Varian 9040 refractive index detector (Varian, St. Helens, Australia) and a Star Chromatography Workstation system control version 5.50 were used.

#### 3.3.3. Phenolic Acids

Ferulic acid (FA), dimers of ferulic acid (di-FA) and trimers of ferulic acid (tri-FA) contents were determined in WEAX and WEAX gel after saponification by RP-HPLC [23,31]. An Alltima C18 column (250 × 4.6 mm) (Alltech associates, Inc. Deerfield, IL, USA) and a photodiode array detector Waters 996 (Millipore Co., Milford, MA, USA) were used. Detection was followed by UV absorbance at 320 nm.

#### 3.3.4. Protein Content

The protein content in the WEAX powder was determined according to the Dumas method [32], using a Leco-FP 528 nitrogen analyzer.

#### 3.3.5. Intrinsic Viscosity and Viscosimetric Molecular Weight

Viscosity measurements were made by determination of the flow times of WEAX solutions in water (from 0.06 to 0.1% w/v). An Ubbelohde capillary viscometer at 25 ± 0.1 °C, immersed in a temperature controlled water bath was used. The intrinsic viscosity ([η]) was estimated from relative viscosity measurements (ηrel) of WEAX solutions by extrapolation of Kraemer and Mead and Fouss curves to “zero” concentration [9,17]. The viscosimetric molecular weight (Mν) was calculated from the Mark-Houwink relationship, Mν = ([η]/k)1/α.

#### 3.3.6. Molecular Weight Distribution

Molecular weight distribution of WEAX was determined by SE-HPLC at 38 °C using a TSKgel (Polymer Laboratories, Shropshire, U.K.) G5000 PWXL column (7.8 × 300 mm). Isocratic elution was done at 0.6 mL/min with 0.1 M LiNO_3_ filtered through 0.2 µm (Whatman). Molecular weigths were estimated after universal calibration with pullulans as standards (P50 to P800). 20 µL of WEAX solution (0.5% w/v filtered through 0.2 µm (Whatman) were injected and a Waters 2414 refractive index detector was used for detection [9].

#### 3.3.7. Fourier Transform Infra-Red (FT-IR) Spectroscopy

FT-IR spectra of dry WEAX and WEAX gel (lyophilized) powder were recorded on a Nicolet FT-IR spectrophotometer (Nicolet Instrument Corp. Madison, WI, USA). The samples were pressed into KBr pellets (2 mg sample/200 mg KBr). A blank KBr disk was used as background. Spectra were recorded between 400 and 4,000 cm^–1^ [17].

### 3.4. WEAX Gelation

A WEAX solution (2% w/v) was prepared in 0.05 M citrate phosphate buffer pH 5.5. Laccase (1.675 nkat per mg WEAX) was added to WEAX solution as cross-linking agent. Gels were allowed to develop for 2 h at 25 °C [9].

### 3.5. Rheological Tests

Small amplitude oscillatory shear was used to follow the gelation process of WEAX solution. Cold (4 °C) WEAX solution (2% w/v) in 0.05 M citrate phosphate buffer pH 5.5 was mixed with laccase and immediately poured on plate-plate geometry (4.0 cm in diameter) of a strain controlled rheometer (Discovery HR-3 rheometer, TA Instruments, New Castle, DE, USA). Exposed edges were recovered with silicone to prevent evaporation. WEAX gelation was started by a sudden increase of temperature from 4 to 25 °C and monitored at 25 °C for 2 h by recording the storage (G’) and loss (G”) moduli. Measurements were carried out at 1.0 Hz frequency and 10% strain. From strain sweep tests, WEAX gels showed a linear behavior from 0.02 to 100% strain. 10% strain was used in all the rheological measurements. The mechanical spectra of gels were obtained by frequency sweep from 0.01 to 10.0 Hz with a 10% strain at 25 °C [9,23].

### 3.6. Structure of Freeze Dried WEAX Gels

WEAX gels were frozen at −20 °C and lyophilized at −37 °C/0.133 mbar overnight in a Freezone 6 freeze drier (Labconco, Kansas, MO, USA). The external structure of the freeze-dried WEAX gel was analyzed with a stereo light microscope (Leica CLS 150 XE Leica Microsystems^®^, Heerbrugg, Switzerland) at a low magnification (10×). The internal structure of freeze-dried WEAX gel was studied by scanning electron microscopy (JEOL 5410LV, JEOL, Peabody, MA, USA) at low voltage (20 kV). SEM image was obtained in secondary electrons image mode.

## 4. Conclusions

Yield of water extractable arabinoxylans (WEAX) from Tacupeto F2001 wheat flour is 0.50% on a dry matter basis (w WEAX/w wheat flour), which is in the range (0.50%–0.68%) reported for French and Canadian wheat varieties, suggesting the potential of this Mexican variety as a source of arabinoxylans. Tacupeto F2001 WEAX present intermediate values of A/X ratio (0.66) and ferulic acid content (0.526 µg/mg) and a FTIR spectral pattern typical for arabinoxylans. This material comprises two populations of polysaccharides with apparent molecular weights in a broad range (50–800 kDa). WEAX is able to form gel in the presence of laccase as shown by dynamic rheometry. Freeze dried WEAX gels present a porous structure constituted by an irregular honeycomb structure. The understanding of Tacupeto F2001 WEAX yield and characteristics can be useful to propose alternative uses of this wheat cultivar. These WEAX gels could be used as microencapsulation systems for bioactive compounds or cells. 
